# Brolucizumab Intravitreal Injections for Wet Age-Related Macular Degeneration: Real-Life Study on a Cohort of Italian Patients

**DOI:** 10.3390/medicina59061110

**Published:** 2023-06-08

**Authors:** Carlo Gesualdo, Settimio Rossi, Clemente Maria Iodice, Francesco Guarino, Mariachiara Petrella, Fabiana Anna D’Agostino, Raffaele Perrotta, Francesca Simonelli

**Affiliations:** 1 Eye Clinic, Multidisciplinary Department of Medical, Surgical and Dental Sciences, University of Campania “Luigi Vanvitelli”, Via S. Pansini, 5, 80131 Naples, Italyfabianadagostino94@gmail.com (F.A.D.); francesca.simonelli@unicampania.it (F.S.); 2 Eye Unit, G. Rummo Hospital, Via dell’Angelo, 1, 82100 Benevento, Italy

**Keywords:** age-related macular degeneration, anti-vascular endothelial growth factor, brolucizumab, central retinal thickness, exudation, intravitreal injection, retinal edema

## Abstract

*Background and Objectives:* To report the real-life Brolucizumab therapeutical outcomes of treatment-naïve and non-treatment-naïve eyes with neovascular age-related macular degeneration (nAMD) and to analyze the incidence of therapy-related adverse events. *Materials and Methods:* A total of 56 eyes of 54 patients diagnosed with nAMD were retrospectively evaluated over a 3-month follow-up. Naïve eyes received a 3-month loading phase, whereas non-naïve eyes were treated with one intravitreal injection + ProReNata scheme. The main outcome measures were best-corrected visual acuity (BCVA) and central retinal thickness (CRT) change. In addition, patients were stratified on the basis of fluid accumulation site, whether intra-retinal (IRF), sub-retinal (SRF), or sub-retinal pigmented epithelium (SRPE), to separately assess the eventual BCVA change in each subgroup. Finally, the incidence of ocular adverse events was evaluated. *Results:* In naïve eyes, a significant improvement of BCVA (LogMar) was observed at all timepoints from baseline (1 month-Mean Difference (MD): −0.13; 2 months MD: −0.17; 3 months MD: −0.24). In non-naïve eyes, a significant mean change was observed at all timepoints, with the exception of 1-month follow-up (2 months MD: −0.08; 3 months MD: −0.05). CRT significantly changed in both groups at all timepoints at a similar pace within the first two months, with naïve eyes displaying a larger overall thickness decrease at the end of the follow-up (Group 1 = MD: −123.91 µm; Group 2 = MD: −110.33 µm). With respect to the location of the edema, a significant BCVA change was observed in naïve patients with fluid in all three sites at the end of the follow-up (SRPE = MD: −0.13 (*p* = 0.043); SR = MD: −0.15 (*p* = 0.019); IR = MD: −0.19 (*p* = 0.041). Non-naïve patients exhibited significant mean BCVA changes only with respect to SR and IR fluid presence (SRPE = MD: −0.13 (*p* = 0.152); SR = MD: −0.15 (*p* = 0.007); IR = MD: −0.06 (*p* = 0.011). One naïve patient experienced acute-onset anterior and intermediate uveitis which completely resolved after therapy. *Conclusions:* Brolucizumab was demonstrated to be a safe and efficient alternative in improving both the anatomical and functional parameters of eyes with nAMD in this small, uncontrolled, series of patients.

## 1. Introduction

Age-related macular degeneration (AMD) is the main cause of blindness in the elderly in industrialized countries [1]. It is possible to distinguish two forms of the disease: the dry or atrophic form (dry-AMD), which is featured by the presence of drusen with a progressive neuroretinal atrophic degeneration, and the wet form (wet-AMD), characterized by the appearance of retinal neovascular membranes (nAMD), either under or above the retinal pigmented epithelium (RPE), which are known to be a well-demonstrated cause of significant and rapid vision loss [1]. Before the introduction of anti-vascular endothelial growth factor (VEGF) agents, thermal laser, intravitreal steroid injections, and photodynamic therapy, or their combination, were considered the standard of care. With the advent of intravitreal therapy with anti-VEGF drugs, the prognosis of wet-AMD has dramatically improved with a significant reduction in legal blindness incidence [2,3,4].

However, many challenges related to the management of patients with wet-AMD still exist [5]. Indeed, it is a condition of growing importance due to both the mean life expectancy increase and its impact in terms of healthcare system costs, given the continuous patient monitoring and the large number of intravitreal drug injections required [5]. Our experience over the years with anti-VEGF agents has led to minimizing the number of injections needed while maximizing visual gains, thereby reducing the patients’ treatment-related physical and psychological burden [5]. 

Therefore, along with adopting standardized regiments requiring less frequent injection protocols, an alternative approach resides in the possibility of developing a more efficient and durable formulation that would alleviate the need for intense therapies.

In this perspective, the introduction of novel and more efficient anti-VEGF drugs is warranted and, in this perspective, the recently approved Brolucizumab (Beovu^®^, Novartis, Basel, Switzerland), a low molecular weight, single-chain antibody fragment targeting high affinity all forms of VEGF-A, was demonstrated to be promising [5,6,7,8]. Indeed, the 26-kDa molecule appears to be much smaller than the currently available drugs, such as ranibizumab, bevacizumab, or aflibercept. In this perspective, higher agent concentrations are allowed to be packed into the standard 0.05 mL volume, with the rationale of increasing both the durability and the efficiency of the molecule within the intravitreal chamber [9].

Two pivotal studies have demonstrated the non-inferiority of Brolucizumab to Aflibercept in treatment-naïve eyes with nAMD with respect to best-corrected visual acuity (BCVA) change at 48 weeks from baseline [10,11]. Additionally, these studies showed a greater reduction in central retinal thickness (CRT), with the possibility of extending dosing intervals up to 12 weeks in more than half of the eyes treated with 6 mg of Brolucizumab after the initial loading phase [10,11]. On the other hand, other studies have reported higher rates of intraocular inflammation (IOI) and retinal vasculitis with or without vascular occlusion in subjects treated with Brolucizumab 6 mg compared to Aflibercept (4.4% versus 0.8%, respectively) [12,13]. 

However, to date, there are only a few short-term, real-life published studies reporting the anatomical and functional outcomes of Brolucizumab therapy showing inconsistent BCVA results compared to the main prospective randomized clinical trials (RCTs), a finding commonly attributed to under-treatment issues and poor therapeutical compliance [14,15,16,17].

Therefore, the aim of this study is to describe, on the largest Italian treated cohort of patients with nAMD analyzed so far in a real-world setting, the therapeutical outcomes of Brolucizumab injections after a 3-month loading phase in treatment-naïve eyes and after one intravitreal injection + ProReNata scheme in non-treatment naïve eyes and to analyze the incidence of therapy-related adverse events.

## 2. Methods

### Participants

In this retrospective observational study, 54 patients (56 eyes) with nAMD showing visual impairment and/or persistence of macular edema on Optical Coherence Tomography (OCT) were enrolled and followed up for 3 months at both the Eye Clinic of the University of Campania “Luigi Vanvitelli”, Naples, Italy and the Eye Unit of “Rummo” Hospital in Benevento, Italy. Table 1 reports demographic and clinical ophthalmic data of the patients included.

This study complied with the tenets of the Declaration of Helsinki and was approved by the Institutional Review Board of the University of Campania “Luigi Vanvitelli”. All patients were provided with an informed consent for participation in the study.

The ocular exclusion criteria considered were previous treatment with Brolucizumab; concomitant ocular pathologies, such as diabetic retinopathy, retinal vascular occlusions, uveitis, vitreoretinal interface pathologies; surgical or parasurgical procedures, such as laser photocoagulation and/or vitrectomy within the last 6 months in the study eye. 

The cohort was categorized into 2 groups: Group 1 (30 Naïve eyes) received intravitreal therapy with Brolucizumab 6 mg (0.05 mL solution) administered according to the HAWK and HARRIER trials guidelines of loading phase (3 intravitreal doses monthly), while group 2 (28 non-naïve eyes) received 1 injection + ProReNata scheme of treatment. 

All participants underwent four examinations (baseline, 1, 2, and 3 months after treatment) that included: BCVA (LogMAR) using Early Treatment Diabetic Retinopathy Study (ETDRS) chart at 2 m, anterior segment slit lamp biomicroscopy, binocular indirect ophthalmoscopy, and Spectral-Domain Optical Coherence Tomography (SD-OCT).

SD-OCT was performed using Maestro 3D OCT-1 (Topcon Corporation, Tokyo, Japan), setting an acquisition protocol that consisted of a 3-D Macula Analysis and a Radial Scan. In all patients, central retinal thickness (CRT) was recorded and assessed by measuring the distance between the vitreoretinal surface and retinal pigment epithelium (RPE) at the foveal center. 

The main outcome measures were BCVA and CRT change over a 3-month follow-up. In addition, patients were stratified on the basis of fluid accumulation site, whether intra-retinal (IR), sub-retinal (SR), or sub-retinal pigmented epithelium (SRPE), to separately assess the occurring BCVA change in each subgroup. Finally, the incidence of ocular adverse events (e.g., vitritis and vasculitis) related to the administration of Brolucizumab was evaluated.

Standard definitions were applied to diagnose and categorize the fluid location, abiding by the latest consensus guidelines, which are herein reported for ease of interpretation [18]. IR edema was considered as fluid accumulation within the retinal architecture, often paired with the formation of cystoid intraretinal spaces. SR fluid was diagnosed as leakage exceeding the local autoregulation capability of removal, which leads to fluid buildup underneath the neurosensory retina. SRPE edema, instead, was identified as a clinically evident detachment of the retinal pigment epithelium monolayer from the underlying Bruch’s membrane. 

Baseline and follow-up images were graded by three independent, experienced investigators (M.P.; F.A.D.; and C.M.I.) and adjudicated by a senior colleague (C.G.)

Continuous variables were reported as mean ± standard deviation (SD), and categorical features were reported as count (frequency). The normal distribution of continuous variables was verified with the Kolmogorov–Smirnov test. Analysis of variance (ANOVA) and post hoc analysis with the Games–Howell test were performed according to the results of the Levene test homogeneity of variance to assess changes for both CRT and BCVA throughout the follow-up. Paired *t*-test was used to assess the mean differences between mean BCVA in each cohort on the basis of fluid site presence (IRF, SR, and SRPE) at 3 months versus baseline. Statistical analysis was performed using IBM SPSS software (IBM Corp. Armonk, New York, NY, USA, version 28.0). Results were considered statistically significant when the *p*-value was <0.05. 

## 3. Results

A total of 56 eyes of 54 patients, 31 males (57.4%) and 23 females (42.6%) affected by wet-AMD, were retrospectively enrolled. The mean age was 77 ± 5.3 years.

No significant differences were found between the two sub-groups with regard to age and gender. 

Table 2 reports a complete list of functional and morphological findings in both naïve and non-naïve patients.

In Group 1 (naïve eyes), a significant (*p* < 0.05) improvement of BCVA was observed at all timepoints from baseline (1 month- Mean Difference (MD): −0.13 LogMAR-Standard Error (SE): −0.14 LogMAR; 2 months-MD: −0.17 LogMAR-SE: −0.14 LogMAR; 3 months-MD: −0.24 LogMAR-SE: −0.14 LogMAR). In Group 2 (non-naïve eyes), a significant mean change was observed at all timepoints except from the 1-month follow-up (2 months-MD: −0.08 LogMAR-SE: −0.11 LogMAR; 3 months -MD: −0.05 LogMAR-SE: −0.11 LogMAR). 

With regard to CRT, it did significantly change in both groups at all timepoints at a similar pace within the first two months, with naïve eyes displaying a larger overall thickness decrease at the end of the follow-up (Group 1 = MD: 123.91 µm—SE: 43.41 µm; Group 2 = MD: 110.33 µm—SE: 21.49 µm). 

**Figure 1:**
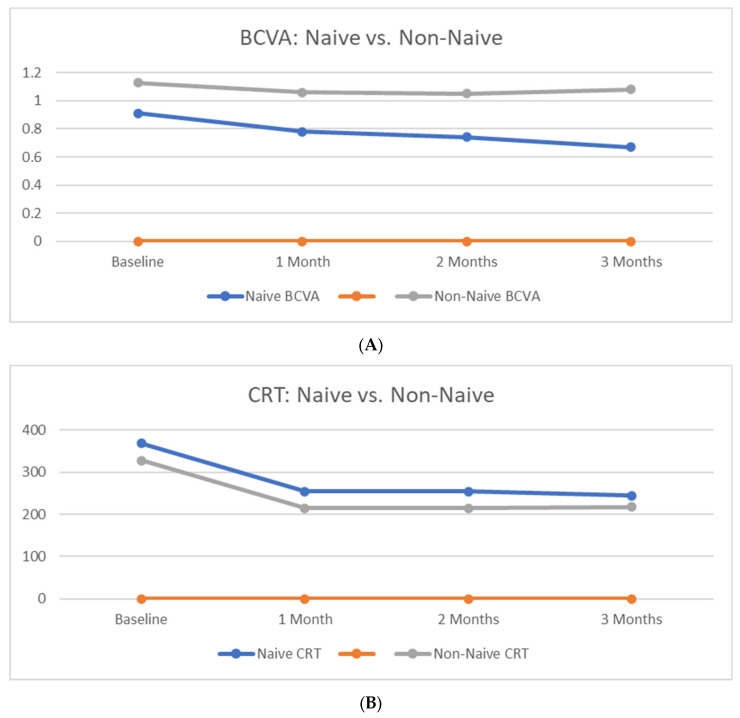
Comparison of BCVA and CRT trends between Treatment-Naïve and Non-Treatment Naïve groups. (**A**): Group 1 (naïve eyes) showed a significant (*p* < 0.05) improvement of BCVA at all timepoints from baseline (1 month-Mean Difference (MD): −0.13 LogMAR-Standard Error (SE): −0.14 LogMAR; 2 months-MD: −0.17 LogMAR-SE: −0.14 LogMAR; 3 months-MD: −0.24 LogMAR-SE: −0.14 LogMAR). Group 2 (non-naïve eyes) displayed a significant mean change at all timepoints except from the 1-month follow-up (2 months-MD: −0.08 LogMAR-SE: −0.11 LogMAR; 3 months-MD: −0.05 LogMAR-SE: −0.11 LogMAR). (**B**): CRT significantly changed in both groups at all timepoints at a similar pace within the first two months, with naïve eyes displaying a larger overall thickness decrease at the end of the follow-up (Group 1 = MD: 123.91 µm–SE: 43.41 µm; Group 2 = MD: 110.33 µm–SE: 21.49 µm).

A line graph comparing the outcome trends between naïve and non-naïve patients is displayed in Figure 1A,B.

In 61.5% (16 eyes) of non-naïve cases, just one intravitreal injection was enough to achieve a significant reduction in the fluid accumulation and both anatomic and functional stabilization of the disease until the end of the follow-up. 

**Figure 2:**
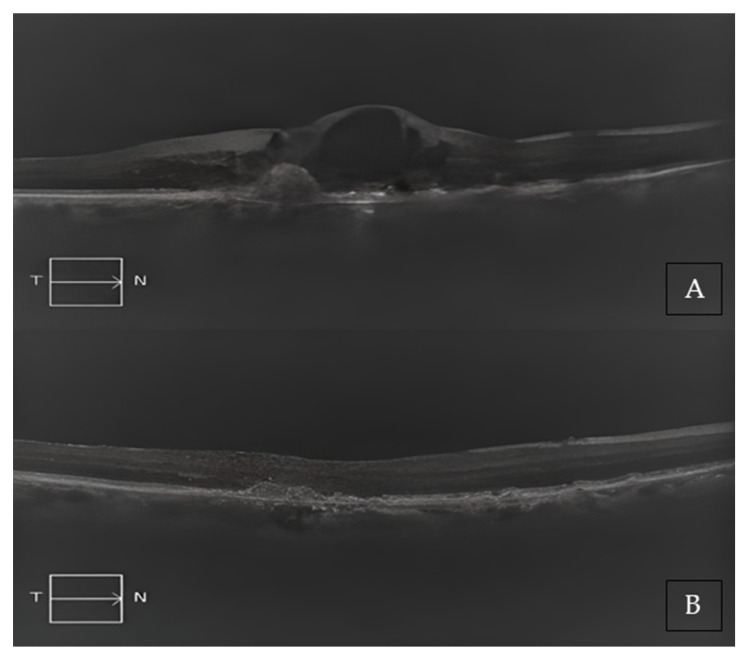
(**A**) Baseline SD-OCT scan acquired with Maestro 3D OCT-1 (Topcon Corporation, Tokyo, Japan) of a non-treatment-naïve patient displaying diffuse intraretinal edema. (**B**) A 4-month SD-OCT scan acquired with Maestro 3D OCT-1 (Topcon Corporation, Tokyo, Japan) displaying subretinal hyper-reflective material and no residual edema.

**Figure 3:**
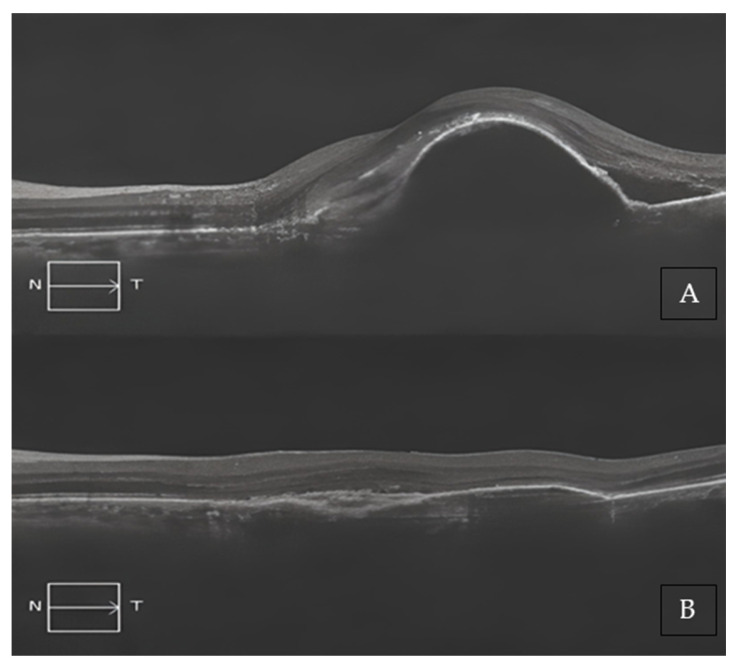
(**A**) Baseline SD-OCT scan acquired with Maestro 3D OCT-1 (Topcon Corporation, Tokyo, Japan) of a treatment-naïve patient displaying a large, pigmented epithelium detachment (PED), and subretinal edema. (**B**) A 4-month SD-OCT scan acquired with Maestro 3D OCT-1 (Topcon Corporation, Tokyo, Japan) displayed a complete reabsorption of the PED and a modest residual sub-RPE hyper-reflective band.

Two representative cases of patients from our cohort, before and after treatment with Brolucizumab, are shown in Figure 2A,B and Figure 3A,B. 

Patients who were treatment-naive and non-treatment-naïve were furtherly allocated into three subgroups, based on the site of fluid accumulation at baseline, to separately assess any occurring BCVA change in each group.

Table 3 reports BCVA change in both treatment and non-treatment-naïve patients based on the site of edema accumulation. With respect to the naïve group, a statistically significant mean BCVA change was observed in patients with fluid presence at all three levels at the end of the follow-up from baseline (SRPE = MD: −0.13 LogMAR-SE: 0.06 (*p* = 0.043); SR = MD: −0.15 LogMAR-SE: 0.03 (*p* = 0.019); IR = MD: −0.19 LogMAR-SE: 0.09 (*p* = 0.041). On the other hand, non-naïve patients exhibited a significant mean BCVA change only with respect to SR and IR fluid presence (SRPE = MD: −0.13 LogMAR-SE: 0.11 (*p* = 0.152); SR = MD: −0.15 LogMAR-SE: 0.02 (*p* = 0.007); IR = MD: −0.06 LogMAR-SE: 0.02 (*p* = 0.011). 

**Figure 4:**
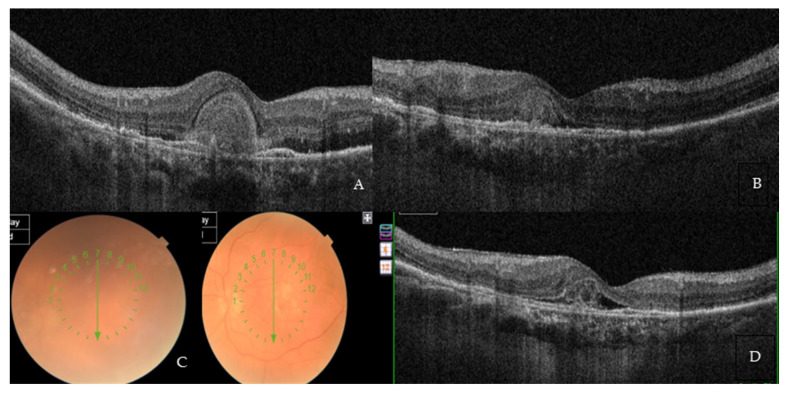
Adverse event of anterior and intermediate uveitis in a 78-year-old treatment-naïve patient. (**A**) Baseline SD-OCT scan acquired with Maestro 3D OCT-1 (Topcon Corporation, Tokyo Japan) prior to the therapy with Brolucizumab displaying hyper-iso-reflective subretinal material distorting the retinal profile, RPE irregularities, and both IR and SR edema. BCVA at this stage was 1.00 LogMAR. (**B**) SD-OCT at the end of the loading phase showed a striking retinal profile restoration with an important reduction in the hyper-iso-reflective subretinal material and both the IR and SR edema. BCVA significantly improved to 0.44 LogMAR. (**C**) Two weeks after the second injection, an anterior and intermediate uveitis with massive vitreous haze occurred with difficulty in exploring the ocular *fundus*. Diffuse inflammatory cells and flare within the anterior chamber, and keratic endothelial precipitates, were also noted. BCVA dramatically dropped to 1.0 LogMAR. (**D**) Three weeks after therapy with high doses of topical and systemic steroids and mydriatic eye drops, a significant improvement in the entire clinical picture was achieved. Eventually, the uveitis totally resolved with a full restoration of BCVA back to 0.44 LogMAR and the complete disappearance of any uveitis clinical finding.

Only one 78-year-old treatment-naïve male with no previous history of systemic inflammatory diseases experienced an acute onset of anterior and intermediate uveitis, which occurred about 15 days after the second loading phase injection. The patient presented with diffuse inflammatory cells and flare within the anterior chamber and keratic endothelial precipitates, along with a significant vitreous haze. His BCVA prior to this event was 0.44 LogMar, which sharply dropped to 1.00 LogMar secondary to the uveitis. The patient was promptly started on a treatment regimen with high doses of topical and systemic steroids and mydriatic eye drops, achieving a significant improvement in the entire clinical picture after only 20 days from the start of therapy (Figure 4A–D). Eventually, the patient fully recovered, as confirmed by the full restoration of BCVA to 0.44 LogMar and the complete disappearance of any overmentioned uveitic clinical feature. Brolucizumab injections were continued as per protocol, and a strict observation was agreed with the patient, who did not show any recurrence or new onset of inflammatory adverse events.

## 4. Discussion

The treatment of wet-AMD has always been challenging throughout the years, aiming at identifying the most efficient treatment alternative to either improve or maintain BCVA over time. The relatively recent introduction of a novel anti-VEGF agent, Brolucizumab, was indeed proven to be a valid therapeutic option [16].

This study assessed and compared the early anatomical and functional changes of naïve patients with wet-AMD treated with a monthly loading phase of Brolucizumab and non-naïve patients who were scheduled to receive one intravitreal injection + ProReNata regime of therapy.

The treatment was shown to be effective, demonstrating a significant recovery of retinal morphology and function in both cohorts. Indeed, BCVA and CRT significantly improved, and fluid accumulated at all levels (IR, SR, and SRPE) was strikingly reduced at the end of the follow-up. It is important to note that naïve patients constantly showed a better improvement in the features considered when compared to the non-naïve group.

In particular, at the end of the follow-up, BCVA and CRT improved considerably more in Group 1 (−0.24 (SE 0.14) and −123.91 (SE 43.41), respectively) versus Group 2 (−0.05 (SE 0.11) and −110.33 (SE 21.49), respectively). 

Previous studies have investigated the Brolucizumab effectiveness in terms of visual acuity increase, for which the reported values in the literature appear to be rather inconsistent with each other. Both the HAWK and HARRIER protocol and the recently published BREW study described significantly lower ranges of BCVA increase after treatment compared to our experience [10,16]. On the contrary, our data supporting the BCVA rise were found to be rather similar to the real-life study published by Bilgic et al., who demonstrated comparable ranges of visual acuity gain with regard to treatment-naïve patients, whereas non-treatment naïve individuals were reported to recover about twice as much as in our cohort [19]. Enriquez et al., instead, reported no significant changes in BCVA at the final follow-up in a cohort of patients who were mainly switched from another anti-VEGF therapy and thus were non-treatment naïve [17]. 

Furthermore, our study confirmed the long-term efficacy of Brolucizumab, especially within the non-naïve group, among which 16 eyes (61.5%) displayed complete reabsorption of both IR and SR fluid accumulation and achieved a substantial stabilization of the retinal exudation after just one single intravitreal injection, thus underlining the usefulness of a therapeutic shift quite early. This finding was consistent with the report of Bilgic et al., who described the exceptional effectiveness of Brolucizumab in drying the macula from fluid in both IR and SR compartments [19]. The authors also claimed that the smaller size of the drug molecule would imply larger concentrations delivered intraocularly that would probably account for its improved efficacy and durability over time [19]. 

However, this consideration may also account for a higher incidence of hypersensitivity-like reactions related to this drug. Indeed, several reports described an association between the Brolucizumab administration and increased propensity towards inflammatory adverse events [13,20]. In our experience, only one treatment-naïve male developed an acute-onset anterior and intermediate uveitis, whereas non-treatment-naïve patients did not demonstrate any adverse event until the last follow-up. Our patient required a treatment regimen of both local and systemic steroids and mydriatic local agents, displaying a complete recovery with no residual deficits noted, either in terms of visual acuity or anatomic changes. The time to development of this adverse event is quite different from past published studies on Brolucizumab-associated inflammatory complications, which suggests that such adverse events are noted rather soon (day 45) in the course of the follow-up [13,20]. The reported incidence of adverse events after Brolucizumab injections were found to be significantly higher in the published literature compared to our analysis. In fact, a cohort of 172 eyes treated with and followed up for 6 months displayed an incidence of 14 cases of intraocular inflammation, all of which completely resolved with no sequelae with adequate and timely treatment [20]. Despite the different rates of complications incidence, it is to note that all reported adverse events responded well to therapy, with our case making no exception. Therefore, it is of pivotal importance highlighting to start treatment upon diagnosis, emphasizing the importance of early recognition of any ocular inflammatory complication.

An interesting observation was made in the recently published PROBE study, in which authors demonstrated the potentialities of a pro-re-nata regime for Brolucizumab therapy [9]. Comparable outcomes in terms of both BCVA and CRT at the end of the 11-month follow-up were observed when compared to both loading dose and treat-and-extend schemes of treatment. Authors claimed that such protocols had been tailored upon molecules (e.g., Ranibizumab of Aflibercept) that were demonstrated to be far less potent and with a far lower molecular concentration than Brolucizumab and, therefore, could be considered obsolete and/or should be revised as far as this new agent is concerned [9]. 

Finally, with regard to the fluid accumulation site at baseline, our study found that after 3 months the IR group displayed complete edema reabsorption in 81% of eyes, while eyes with SRF in 95% and SRPE in 92%. In the Hawk and Harrier trials, the ratio of eyes with IR and/or SR edema at week 96 was strikingly lower in the Brolucizumab treatment arm compared to the Aflibercept arm [10,11]. Matsumoto et al. described a cumulative complete absence of any intraretinal, subretinal, and sub-RPE fluid in 47.2% of eyes after 1 month from Brolucizumab injection, in 86.1% after 2 months, and 94.4% after 3 months [21]. Montesel et al. retrospectively observed IRF and SRF presence at baseline in 63% and 89% of eyes, respectively, which both shrunk to 16% at the last follow-up [22]. The same trend was confirmed by Toto et al. for IR, SR, and SRPE edemas [15].

Considering the BCVA improvement stratified to fluid accumulation site, instead, the treatment-naive IR group demonstrated better outcomes versus the non-naïve group, while eyes with baseline SRPE displayed significant positive changes only for treatment-naïve individuals. As for the two SR groups, instead, they both showed comparable inter-group results. The possible reasons to which ascribe better trends found in the treatment-naïve compared to the non-treatment naïve group are probably to be attributed to the fact that the second subgroup of patients, despite receiving an intensive protocol with previous anti-VEGF drugs, showed no or little response to those agents, which led to drug switching. The therapeutic refractory course of the disease led, in turn, to a compromission of the local cellular homeostasis that reflected worse functional gain compared to the efficiently treated naïve patients. 

The main limitations of the present study are associated with its retrospective, real-life design, which, by its nature, can include low internal validity, lack of quality control surrounding data collection, and susceptibility to multiple sources of bias for comparing outcomes. The relatively small cohort size and the short-term follow-up, and the absence of a control group, should also be taken into account.

## 5. Conclusions

In conclusion, the relatively recent introduction of Brolucizumab has given rise to new therapeutic possibilities for patients with wet-AMD. The drug was demonstrated to be effective in improving both the anatomical and functional parameters and was shown to be successful at obtaining a dry macula. Moreover, its efficiency was also demonstrated in terms of the number of injections needed to achieve such optimal outcomes compared to previous anti-VEGF agents. As far as we are concerned, although the overmentioned limitations related to the study design, this current analysis is still an accurate portrayal of the current capabilities of intravitreal therapy for neovascular AMD with Brolucizumab and the challenges associated with its real-world application as opposed to a randomized trial setting. This agent was indeed effective in treating both treatment-naïve and non-naïve patients, allowing significant improvement in both BCVA and CRT. 

Nevertheless, more studies with longer follow-ups on larger sample sizes are warranted on this topic to better manage and standardize the therapeutical approach, to better understand the real treatment outcomes, and to better investigate the long-term effects and the adverse reactions of this promising novel anti-VEGF agent.

## Figures and Tables

**Table 1:** Demographic and clinical ophthalmic data of the patients enrolled in the study.

Characteristics			Naive	Non-Naive
Age—Mean (SD)		Years	Years	Years
		77 (±5.3)	76 (±5.2)	78 (±4.3)
Gender	Male	31 (57.4%)		13 (61.9%)
			18 (54.5%)	
	Female	23 (42.6%)	15 (45.5%)	8 (38.1%)

N. Eyes			30 (53.6%)	26 (46.4%)
Lens status		15 (28.5%)		
	Phakic			
	Pseudophakic	39 (71.5%)		


SD: standard deviation; N.: number of.

**Table 2:** Functional and morphological data of naïve and non-naïve patients.

		BCVA	*p*	CRT	*p*
Naive	B.	0.91		367.82	
	1MO	−0.13 (SE 0.14)	0.04	−113.50 (SE 41.26)	0.05
	2MO	−0.17 (SE 0.14)	0.02	−114.08 (SE 43.03)	0.04
	3MO	−0.24 (SE 0.14)	0.04	−123.91 (SE 43.41)	0.03
Non-naive	B.	1.13		327.73	
	1MO	−0.07 (SE 0.11)	0.01	−112.80 (SE 19.75)	<0.001
	2MO	−0.08 (SE 0.11)	0.01	−112.80 (SE 19.70)	<0.001
	3MO	−0.05 (SE 0.11)	0.15	−110.33 (SE 21.49)	<0.001

MO: months; B.: baseline; BCVA: best-corrected visual acuity; CRT: central retinal thickness; SE: standard error; *p*: *p*-value.

**Table 3:** Best-corrected visual acuity changes in treatment-naive and non-treatment-naïve patients stratified by the site of edema accumulation.

		BCVA	*p*
Naive	B.	0.91	
	IR	−0.19 (SE 0.09)	0.04
	SR	−0.15 (SE 0.03)	0.02
	SRPE	−0.13 (SE 0.06)	0.04
Non-naive	B.	1.13	
	IR	−0.06 (SE 0.02)	0.01
	SR	−0.15 (SE 0.02)	0.01
	SRPE	−0.13 (SE 0.11)	0.15

B.: baseline; BCVA: best-corrected visual acuity; IR: intra-retinal; SR: sub-retinal; SRPE sub-retinal pigmented epithelium; SE: standard error; *p*: *p*-value.

## Data Availability

The datasets used and/or analyzed during the current study are available from the corresponding author upon reasonable request.
